# Draft Genome Sequence of Bacillus pumilus SCAL1, an Endophytic Heat-Tolerant Plant Growth-Promoting Bacterium

**DOI:** 10.1128/genomeA.00306-18

**Published:** 2018-05-03

**Authors:** Tehmeena Mukhtar, Muhammad S. Afridi, Robyn McArthur, Jonathan D. Van Hamme, Francois Rineau, Tariq Mahmood, Amna, Sumaira, Muhammad Zahid, Abdul Salam, Muhammad N. Khan, Fawad Ali, Shehzad Mehmood, Naila Bangash, Hassan J. Chaudhary

**Affiliations:** a Department of Plant Sciences, Quaid-i-Azam University, Islamabad, Pakistan; b Department of Biological Sciences, Thompson Rivers University, Kamloops, British Columbia, Canada; c Center for Environmental Sciences, University Hasselt, Hasselt, Belgium; d Department of Genetics, Hazara University Mansehra, Khyber Pakhtunkhwa, Pakistan; e Department of Biotechnology, Quaid-i-Azam University, Islamabad, Pakistan; f Department of Biochemistry, Abdul Wali Khan University Mardan, Mardan, Pakistan; g Department of Microbiology, Quaid-i-Azam University, Islamabad, Pakistan

## Abstract

Bacillus pumilus strain SCAL1 is an endophytic, thermophilic plant that was isolated from the leaf of a plant, Solanum lycopersicum L., in Sindh, Pakistan. B. pumilus strain SCAL1 has usually exhibited high resistance to environmental stresses, with a growth temperature ranging from 30 to 60°C. An approximately 3.75-Mb draft genome was assembled into 68 contigs.

## GENOME ANNOUNCEMENT

Bacillus pumilus forms endospores and naturally occurs in roots, leaves, seeds, stems, needles, twigs, and bark of various plant species (1–4). In this context, plant growth-promoting bacteria improve plant growth either (i) directly, through production of phytohormones, ammonia, siderophores, or enzymes (ACC deaminase and catalase), or by providing nitrogen, phosphate, or iron, or (ii) indirectly, by antagonistic mechanisms which protect plants from pathogens and insects. Certain endophytes generate induced systemic tolerance (IST), through which plants are protected from abiotic stressors and allowed to attenuate their negative effects (3–6). Bacterial spores show resistance to radiation, desiccation, and hydrogen peroxide treatment (7, 8).

After sample collection, surface sterilization was done (1). Strain taxonomy was based on 16S rRNA gene sequencing (2, 7, 9). Two milliliters of cell culture broth was taken and centrifuged at 12,000 *× g* for 5 minutes. RNA-free genomic DNA was extracted with an Invitrogen PureLink genomic DNA minikit and quantified using a Qubit double-stranded DNA (dsDNA) HS assay kit (Life Technologies, Inc., Burlington, ON). Adaptor-ligated DNA was size selected to 480 bp on a 2% E-Gel SizeSelect agarose gel, and Agencourt MAPure XP beads (Beckman Coulter, Mississauga, ON) were used for purification. Library dilution factor was determined using an Ion Library quantitation kit prior to amplification and enrichment with an Ion PGM template OT2 400 kit on an Ion OneTouch 2 system. The enriched Ion Sphere particles were quantified using an Ion Sphere quality control kit prior to sequencing on a 316 version 2 chip with an Ion PGM 400 sequencing kit on an Ion Torrent PGM. Sequencing generated 590,303 reads (mean length, 256 bases) and 151 Mb of data (>127 million Q20 bases) in Torrent Suite 4.4.3. Further, assembly was performed using SPAdes 3.1.0 (10, 11) with trimming by custom script Trim_SPAdes into 68 contigs greater than 500 bp, giving a consensus length of 3,743,482 bp at 21× coverage (largest contig, 371,528 bp; *N*_50_, 112,210 bp) with a GC content of 41.4%. The Rapid Annotations using Subsystem Technology (RAST) server (8, 12, 13) was used to find closely related strains before using progressiveMauve version 2.3.1 (14) to reorder scaffolds with the use of B. pumilus SAFR-032 (NCBI reference sequence NC_009848) (15) as a reference. Open reading frame (ORF) prediction and gene annotation were done by using the NCBI Prokaryotic Genome Annotation Pipeline (PGAP) pipeline (16, 17). Gene search and annotation were performed for all contigs longer than 500 bp using the NCBI Prokaryotic Genomes Automatic Annotation Pipeline and the RAST server (8). The SEED viewer was used for assignment of predicted genes to functional categories (18). The RAST server predicted 3,327 coding sequences (CDS) with a total of approximately 3,802 genes, 400 pseudogenes, 57 tRNAs, and 1 noncoding RNA (ncRNA) gene and 17 coded rRNAs (5S, 16S, and 23S). Genome-wide phylogenetic analysis of various Bacillus species compared to the B. pumilus SCAL1 genome showed that it clusters closely toB. pumilus SAFR-032. Genome analysis revealed the genes for heat shock resistance, hydrocarbon metabolism, heavy metal tolerance, biofilm formation, and siderophore and IAA biosynthesis.

### Accession number(s).

This whole-genome shotgun project has been deposited at DDBJ/ENA/GenBank under the accession number LFIZ00000000 (version LFIZ01000000).
