# Correction for Mukhtar et al., “Draft Genome Sequence of Bacillus safensis SCAL1, an Endophytic Heat-Tolerant Plant Growth-Promoting Bacterium”

**DOI:** 10.1128/MRA.01397-19

**Published:** 2019-12-12

**Authors:** Tehmeena Mukhtar, Muhammad S. Afridi, Robyn McArthur, Jonathan D. Van Hamme, Francois Rineau, Tariq Mahmood, Amna, Sumaira, Muhammad Zahid, Abdul Salam, Muhammad N. Khan, Fawad Ali, Shehzad Mehmood, Naila Bangash, Hassan J. Chaudhary

**Affiliations:** a Department of Plant Sciences, Quaid-i-Azam University, Islamabad, Pakistan; b Department of Biological Sciences, Thompson Rivers University, Kamloops, British Columbia, Canada; c Center for Environmental Sciences, University Hasselt, Hasselt, Belgium; d Department of Genetics, Hazara University Mansehra, Khyber Pakhtunkhwa, Pakistan; e Department of Biotechnology, Quaid-i-Azam University, Islamabad, Pakistan; f Department of Biochemistry, Abdul Wali Khan University Mardan, Mardan, Pakistan; g Department of Microbiology, Quaid-i-Azam University, Islamabad, Pakistan

## AUTHOR CORRECTION

Volume 6, no. 18, e00306-18, 2018, https://doi.org/10.1128/genomeA.00306-18. Page 1: The article title should read as given above.

Pages 1 and 2: The bacterial strain should be identified throughout as Bacillus safensis rather than Bacillus pumilus. In August 2018, NCBI staff performed an average nucleotide identity (ANI) analysis and determined that the organism name should be changed.

